# The ‘other’ in patterns of drinking: A qualitative study of attitudes towards alcohol use among professional, managerial and clerical workers

**DOI:** 10.1186/1471-2458-12-892

**Published:** 2012-10-23

**Authors:** Jonathan Ling, Karen E Smith, Graeme B Wilson, Lyn Brierley-Jones, Ann Crosland, Eileen FS Kaner, Catherine A Haighton

**Affiliations:** 1 Department of Pharmacy, Health & Wellbeing, University of Sunderland, Sunderland, SR1 3SD, UK; 2 Institute of Health & Society, Newcastle University, Newcastle upon Tyne, NE2 4AX, UK

**Keywords:** Alcohol, Focus groups, Public health, Norms

## Abstract

**Background:**

Recent evidence shows that workers in white collar roles consume more alcohol than other groups within the workforce, yet little is known about their views of drinking.

**Methods:**

Focus groups were conducted in five workplaces to examine the views of white collar workers regarding the effect of alcohol use on personal and professional lives, drinking patterns and perceived norms. Analysis followed the method of constant comparison.

**Results:**

Alcohol use was part of everyday routine. Acceptable consumption and ‘excess’ were framed around personal experience and ability to function rather than quantity of alcohol consumed. Public health messages or the risk of adverse health consequences had little impact on views of alcohol consumption or reported drinking.

**Conclusions:**

When developing public health alcohol interventions it is important to consider the views of differing groups within the population. Our sample considered public health messages to be of no relevance to them, rather they reinforced perceptions that their own alcohol use was controlled and acceptable. To develop effective public health alcohol interventions the views of this group should be examined in more detail.

## Background

Increasing alcohol consumption and the associated health, social and economic harms are key public health concerns [1-4]. While some research has investigated the relationships between job status and alcohol intake [5] and between alcohol consumption, work-related stress and occupational role [6,7], people in higher socio-economic groups are generally under-represented in alcohol research. Recent statistics show however that households with an adult working in a managerial or professional capacity have the highest proportion of alcohol consumption in the previous seven days [8]. Similarly, adults in managerial and professional households are significantly more likely than those in routine and manual households to have had an alcoholic drink on five or more days in the previous week. No research has yet examined how alcohol is viewed by this largely unconsulted section of the population: those in occupations that are managerial, supervisory, clerical or professional – frequently referred to as white collar workers (contrasting with ‘blue collar’, unskilled or manual workers).

Alcohol holds an established role within British culture where it is associated with socialising, pleasure, celebration and escape from pressure [1,4]. While the majority of alcohol users are sensible drinkers, alcohol use outside socially defined acceptable parameters is viewed negatively and is also strongly associated with illness and crime [9,10]. Recent public health policies have focussed upon young people, binge drinking and the socially visible consequences of problematic drinkers, largely disregarding the harmful health and social effects of average alcohol consumption over time [1,3,9]. The UK Government’s latest alcohol strategy does acknowledge the health impact of alcohol use; however its focus remains *“turn[ing] the tide against irresponsible drinking”*[4], p4].

In the UK there has been a shift away from drinking within leisure premises and an increase in home drinking [1,4], where the majority of drinking now takes place [11]. Home drinking is generally portrayed as safe and responsible despite being typically uncontrolled and unregulated [11]. At a population level, there is increasing awareness of the daily guidelines for responsible drinking [4,12], however these are not applied consistently to personal behaviour [1,13]. Higher levels of alcohol consumption have become normalised [1,14] and many people now adhere to a personally-interpreted definition of moderate drinking that could put them in danger of short- and long-term negative consequences [12]. Almost a quarter of the population report regularly drinking in excess of current guidance, an overwhelming majority of whom do not think they are causing any risk to their long term health and, unlike people who smoke - most of whom would like to quit - less than a fifth of those regularly drinking in excess of the recommended guidance want to drink less [4]. This presents a significant and growing health burden [11,15] and has important implications for future public health approaches around alcohol use [9].

Accessing individuals in the workplace provides an important opportunity to increase understanding of the views underpinning health behaviours of working people, who form a significant proportion of the adult population. This study explored white collar workers’ views of alcohol use. Going beyond a discussion of consumption, we sought to develop an understanding of how public health alcohol messages were viewed, as well as exploring contextually the role of alcohol within the personal and professional lives of white collar workers.

## Methods

### Participants

An opportunity sample of 49 people (17 male, 32 female) participated. Ages ranged from 21 to 55. All participants were working full-time - at least 35 hours per week - in managerial, supervisory, clerical or other professional roles. Participation was voluntary and staff were recruited on behalf of the research team by a co-ordinator within each workplace. Co-ordinators were typically from human resources or a health improvement/health and safety officer. Lunch was provided and a £5 voucher offered to each participant in thanks.

### Data collection

Focus groups were held in five workplaces during employee lunch breaks and were attended by employees from that organisation only.

•  Focus groups 1 and 2 - local government offices. Focus Group 1 consisted of 9 females with ages ranging from 21 to 55. Focus Group 2 was composed of 8 females and 1 male, aged from 25 to 55.

•  Focus group 3 – a private sector chemical storage company. This group consisted of 1 female and 9 males, aged from 25 to 55.

•  Focus group 4 - a prison. The focus group consisted of 7 females and 4 males who ranged in age from 22 to 54.

•  Focus group 5 – a tax office. The focus group consisted of 7 females and 3 males who were aged from 36 to 51.

Each focus group was facilitated by two researchers. Prior to focus groups, participants were given an overview of the research aims to enable provision of informed written consent. At the start of each group ground rules were established to observe confidentiality and facilitate mutual respect. Participants were advised that the facilitators were not seeking personally sensitive information, such as the quantity or frequency of alcohol consumption. Open-ended questions were posed around loosely constructed themes enabling members of the focus groups to raise issues of significance to them, as well as exploring areas of agreement and disagreement. Focus group facilitators worked from a flexible schedule of open-ended questions. These questions were continually reframed in light of emerging concepts based on views related to four themes related to drinking:

•  lifestyle behaviours

•  drinking in the home

•  variations in consumption through the week

•  the effect of drinking on work

Focus groups lasted between 40 and 75 minutes and were audio recorded and transcribed verbatim, with personal information anonymised prior to analysis.

### Data analysis

Data were analysed using the method of constant comparison. Constant comparison requires that data be simultaneously encoded and analysed to enable hypothesis discovery and theory generation [16]. All data were analysed for classification into initial categories, with thematic categories being compared both within and across transcripts. Responses from earlier groups informed discussions in later groups. Comparison continued until saturation. Categories were subsequently integrated into a set of higher level concepts in a process of initial theory generation. Data were first analysed independently by focus group facilitators, and later by other members of the research team who had not been involved in data collection. Regular research team meetings were held to discuss issues arising and to ensure analytic rigor.

## Results

Within all focus groups, drinking alcohol was seen as a reward after fulfilling work commitments and family obligations, and as a way to unwind, alleviate stress or socialise. As discussions developed, socially-acceptable norms were continually negotiated. These norms were constructed around generic drinking behaviours rather than personal consumption (‘you’ and ‘they’ rather than ‘I’) and language and terminology were used to enhance statements, invite consensus from other group members, and identify deviance from these norms.

Three themes emerged:

•  perceptions of harmful, unacceptable or problematic alcohol use as ‘the other’

•  normalisation of alcohol use when perceived to be controlled and harm free

•  the ability to function as an endorsement of acceptable alcohol use

### Perceptions of harmful, unacceptable or problematic alcohol use as ‘the other’

Focus groups considered unacceptable or problematic use of alcohol to be associated with long-term, heavy and binge drinking. Perceptions of excessive alcohol intake were assessed in relation to how a person looks and behaves rather than the quantity or frequency of consumption. Clear distinctions were made between personal use of alcohol and that of *‘others’* by using broadly constructed stereotypes of individuals with complex needs, those using alcohol as a coping mechanism, or those outside their own peer groups such as *‘young people’.*

"The people who are predominantly doing it [drinking problematically] are in a society and a culture where it just becomes the norm; they don’t know any different - they can’t get out of it - but then you are moving into a situation where you are looking at far more than just the alcohol side of things. (Female, Focus group 1)"

The stigma associated with alcohol problems was further highlighted in relation to seeking information or support. If colleagues or peers were experiencing problems with alcohol it was felt likely that there would be an underlying causal factor. Someone experiencing alcohol-related problems was viewed as less worthy of sympathy than someone experiencing other health problems. It was felt that experiencing problems with alcohol would provoke shame, a reluctance to discuss problems with colleagues, especially managers, and an avoidance of any support or services within the workplace.

"I was just thinking for me personally… if I thought my drinking was a problem, the last thing I would want to do then is admit that within work, because then that becomes a double problem: I've got my drinking that's a problem outside of work, and it's impacting on my work and having to admit that. (Female, Focus group 5)"

Personal drinking was viewed as something group members *choose* to do, not something they *need* to do. The idea that drinking could have an adverse impact on their health was refuted. With the exception of messages around drinking and driving, no connection was made between personal behaviour and current public health messages or potential risks to health.

"Just speaking for myself, I am fully aware of all the information and fully aware of what I should be doing and what I shouldn’t be doing and how I should drink and when I should drink, but I am making a choice. I've seen all the education, I don’t think I drink excessively but if you put me on a scale according to the Government I am off the scale but, I feel fit, healthy.. (Male, Focus group 3)"

When discussions focused upon the more severe health and social order impact of alcohol use, negative language and the construction of stereotypes were used to dissociate this from personal ‘acceptable’ use. ‘*Young people’* and those for whom it was *‘too late’* were commonly identified as *‘those people’* for whom public health messages should be targeted.

"I know what you mean yeah drinking to excess-when you see these young teenagers on the streets can’t walk, sort of like collapsed in a heap cos they've drank that much. (Female, Focus group 5)"

Throughout the focus groups, socially acceptable norms were continually negotiated among participants. As such, these can be considered as some of the factors underlying the mechanisms controlling alcohol consumption. In some groups when comments or anecdotal incidents were recounted around alcohol use that fell outside the negotiated or anticipated ‘group norm’ this line of discussion ceased instantly and in some groups those participants raising these issues fell quiet for a period of time.

…the safe drinking limit 2–3 units a day for women, which I suppose is the government sensible recommended daily drinking allowance, however to me and you that's like a couple of glasses of wine, and for me yeah that's sensible but I would have another couple until I got like bladdered, do you know what I mean so, it's…

-I think it's a lot. I think it's a lot

-What's a lot?

-To say 2–3 units a day,

-It does sound a lot doesn’t it?

- I would be worried if I was having 2–3 units a day, every day, personally.

- That's just a glass of wine,

- And it's a unit, isn’t necessarily a big glass of wine, so it's a small wine

*- I would be worried if I was having that every day anyway.* (Interaction between two females, Focus group 1)

Where issues were discussed which were considered to fall outwith the socially defined acceptable norm, negative terms such as ‘shouldn’t’, ‘you just wouldn’t’, ‘that just doesn’t happen anymore’ and ‘isn’t it’ were used along with vocal emphasis to invite group consensus greatly influenced the direction of discussions. This created an environment where only the more confident individuals might have felt comfortable raising opposing views and was particularly apparent when talking about lunchtime drinking, drink driving and smelling of alcohol while at work.

### Normalisation of alcohol use when perceived to be controlled and harm free

Home drinking was considered to be widespread, socially acceptable and convenient. Focus groups reported that they now drank less often within leisure premises. Low cost and easy availability of alcohol and avoidance of drink driving were highlighted as key factors underpinning this change; alcohol was cited as a standard item on supermarket shopping lists. Home responsibilities were also acknowledged as influencing how participants drank and many described completing household chores and family routines before eating a meal with wine or settling down, with a drink, to relax. Drinking was considered a socially acceptable form of relaxation and a marker of the transition from work or parental responsibilities, to ‘me time’.

"I drink one, because I've had a stressful day at work, two because I've had a stressful day at home. I have four children so what I do is children things and so then when I do get the kids off to bed sometimes it's nice to have a drink because it actually makes you feel like an adult again…. Like I say alcohol at home is cheap, [at] your supermarkets you can get a nice bottle of wine for £5 you go to a pub or restaurant and you’re paying £20 for it, so it's more accessible and it's easier and it's more comfortable in your own environment. (Male, Focus group 3)"

Alcohol use was considered part of everyday routine but something which does not interfere with other aspects of life. Alcohol consumption on nights during the working week was considered commonplace and acceptable provided that work and other responsibilities will be fulfilled the following day. Such drinking was interpreted as essentially harm free, despite discussions identifying consumption often greater than that currently recommended for responsible alcohol use. Drinking was also associated with social rituals, such as wine with meals or beer while watching sport.

"I think you probably drink more if you are at home simply because you haven’t got the chew of going up to the bar to buy another drink and losing your seat and all that goes with it. At home you just, I would presume, just sit with the bottle next to you. (Male, Focus Group 2)"

It was acknowledged that heavier drinking sessions took place; however these were associated with time off work. They were typically pre-planned, usually going out in groups of friends, often of the same gender, with the predetermined aim of drinking what were considered large quantities. Contingency plans for transport home, childcare and recovery time were also typically prearranged. The preplanning and infrequent nature of these sessions reinforced perceptions that personal alcohol use was controlled and acceptable.

Current drinking behaviours were not considered to have a negative impact upon short or long-term health. Alcohol use was moderated according to personal awareness and previous experience, with behaviour based upon how group members felt while drinking, how they anticipated they would feel the next day and commitments they would have to fulfil while potentially impaired. The amount of alcohol which constitutes *‘too much’* was evaluated in terms of a person’s size, metabolism and overall state of health. Alcohol intake was gauged according to perceived tolerance levels, rather than according to an absolute number of units of alcohol. Perceptions of excessive alcohol intake in particular, were based on past experience.

"I think you just know how you feel and you have to judge it like that because you couldn’t read every bottle in every bar in every pub so I think people tend to just go on how they feel. (Male, Focus Group 5)"

Where adverse effects were considered these were only discussed in terms of coping with a hangover and the inconvenience of lost time while unwell.

"I think more people care about what they look like on the outside than the inside so if you are not putting a lot of weight on I don’t think people care that much unless you start weeing blood or something. (Male, Focus group 3)"

### The ability to function as an endorsement of acceptable alcohol use

The ability to function at work and act as a responsible adults were considered to be crucial indicators that drinking remains within acceptable levels. The implication was that, as the members of the focus groups were able to maintain employment in skilled roles, they were *by definition* drinking in a way that cannot be hazardous or harmful. One participant did however highlight the tendency for *the ‘functioning alcoholic’* to be overlooked within society.

Within all focus groups *‘smelling of alcohol’* while at work was considered negative and stigmatising; an unprofessional way to present oneself which will lead to a loss of professional credibility. Lunchtime drinking was considered taboo, very much a thing of the past.

Although awareness of the recommended guidelines for responsible drinking exist, little notice was taken of them and there was much confusion as to what constitutes ‘a unit’ and how this equates in terms of drinks consumed. Guidelines were discredited and considered a form of *‘nanny stateism’*.

"Well it's been discredited anyway hasn’t it recently, because I mean the last thing I read about units etc., is that this man had just decided all by himself what a unit was and that then became the recommended guidance. So really it wasn’t backed up by anything particular, it was just this bloke thought 'that sounds about right' and after that it was given out as recommended guidance. (Female, Focus Group 2)"

Across all focus groups, driving was identified as the greatest factor influencing drinking behaviour, being integral to professional and personal lives. Drinking and driving were consistently expressed as unacceptable and losing the ability to drive was seen as impacting upon social status and income, disrupting routine life and stigmatising self and family.

"The driving bit stops me because I hate to not have my car and that is the big thing with me so that stops me from drinking when I know I am out the next day in the car. (Male, Focus group 4)"

Many members of the focus groups had children and other family commitments and while alcohol use was identified as routine, responsibilities were prioritised. Alcohol use was typically negotiated with partners around responsibilities, or took place after commitments were fulfilled; again reinforcing perceptions that such alcohol use was controlled and acceptable.

## Discussion

People working in white collar occupations are under-represented within alcohol research. In this study we found that they considered their alcohol use to be positive and within personal control. A socially-constructed concept of acceptable use was widely agreed, this was both justified and reinforced by the construction of stereotypes of the deviant ‘other’ to describe less acceptable alcohol use. This supports existing evidence which suggests that alcohol use that conforms to socially-defined parameters is an established element of British culture [1,10,14].

A cultural shift was reported away from drinking alcohol in leisure premises and drinking at lunchtimes or directly after work to less public drinking within the home, a pattern identified by Foster et al. [11]. We found that while drinking was still associated with sociability, home drinking was described as more convenient and more affordable than going out, especially for those with family responsibilities. Importantly, however, home drinking is typically less regulated than drinking in licensed premises and while there was some awareness of recommended guidelines for alcohol consumption, there was widespread confusion as to how these translate into drinks consumed.

Despite discussions indicating that reported alcohol use exceeds recommended guidelines for both amount and frequency of consumption, there was no acknowledgement that personal alcohol use - almost invariably considered by participants to be moderate - can incur any harmful health or social consequences. Notions of moderate drinking are underpinned by normative explanations such as the presumed behaviour of others and ‘acceptability’ and ‘excess’ are judged by the ability to function and act responsibly. This supports recent work that concluded that the UK population has become habituated to high levels of alcohol consumption [14]. As people tend to overestimate and exaggerate the drinking of their peers [17], this is likely to have considerable health consequences and could present a significant challenge for public health and treatment services [11,15]. This issue is of particular concern as frequent heavy drinking has been found to have more significant consequences for health than episodic binge drinking [10,18].

In recent years, public health policies have largely focused upon young people and the social and criminal disorder associated with alcohol use [1,9]. Our study has found that these messages had some unintended consequences as focus groups readily identified alcohol problems as being the domain of young people and disordered drinking. Recent public health alcohol messages have therefore not only failed to resonate with white collar workers, they have actively *reinforced* their view that their own alcohol use was problem-free. The ‘problem drinker’, or the individual likely to experience health problems in later life, was not viewed as the stay-at-home evening wine drinker, able to drive when required, provide and care for their family and function effectively within the workplace. Therefore, with the exception of drink driving campaigns, recent public health alcohol messages have failed to impact on the behaviour of a large audience, with potentially significant financial and public health cost implications. Having a better understanding of how ‘acceptable’, ‘moderate’ and ‘problematic’ drinking are viewed among differing groups within the population can help focus the development of future public health alcohol interventions. This is of particular relevance in light of recent findings showing that increased alcohol consumption established in early and mid life are likely to be continued into later life [1,19].

### Strengths and limitations

This is one of the first studies to investigate drinking patterns among white collar workers. The qualitative approach, using focus groups to collect data, allowed themes relating to views of alcohol use to emerge. This study helps reveal the meanings attached to alcohol use by white collar workers and identifies resistance to public health messages. The relative consistency of data across all focus groups indicates that their norms may reflect a wider cultural discourse independent of group variables such as the presence of ‘strong personalities’ in the group or the nature of the business of the workplace involved.

The context of focus groups - in the workplace, with colleagues - is likely to have impacted upon willingness to express views on alcohol and alcohol use that were felt to be counter to group expectations. Discussions within groups constitute public discourse and reactions and expectations shaped how conversations proceeded: where a contribution was anticipated as likely to be questioned or questionable, there was sometimes an attempt to reinforce the validity of statements by asserting an unprovable extreme – ‘everybody drinks’ or ‘the vast majority’, usually with some emphasis. This is likely to have influenced subsequent contributions by others [20] although social norms will have predominated, which was the central aim of this study.

### Implications for practice and future research

The shift to home drinking reported in this study has ramifications for the future development of public health alcohol interventions particularly as focus group members consider themselves to be moderate drinkers. This study has shown that convenience and affordability are factors underpinning the shift to home drinking - where consumption is largely unregulated. Current proposals to address alcohol use in the UK at a population level by curbing the availability of cheap alcohol include minimum unit pricing and the ‘Responsibility Deal’ with the alcohol industry [4]. As with most products, price, availability and available income do influence purchasing behaviour [14], however little is known about whether the UK population will support moves to increase alcohol prices and it is possible that people will absorb increased costs in order to maintain levels of alcohol consumption by sacrificing other expenditure. While there is a growing body of evidence to support these approaches [21,22], such policies may widen public health inequalities and, if cheaper and stronger forms of alcohol or other intoxicating substances are chosen instead, exacerbate existing drug and alcohol problems.

The latest UK Government’s Alcohol Strategy [4] proposes a review of the guidelines for responsible drinking. Our study has shown that while there was awareness among white collar workers of current guidelines, they found them confusing and not readily translatable into the drinks they consume. As well as reviewing the guidance it would be prudent to also examine how messages are communicated to different segments of the population.

This study found driving to be a key factor underpinning reported alcohol behaviour. Focus groups considered current public health messages relating to the responsible use of alcohol to have little or no relevance to them with the exception of campaigns against drinking and driving. Emslie et al. [1] similarly found that driving was a key reason (whether valid or not) offered by respondents to resist friendly pressure to drink. The failure of existing public health messages to engage this group indicates that a different approach should be considered. The latest UK Government’s Alcohol Strategy [4] outlines a commitment to increase the scope and funding for ‘Drinkaware’ to best direct interventions to specific target groups within the population. Our research has shown that a campaign interweaving health messages with those around drink driving would potentially resonate with this group for whom other campaigns have failed to impact.

Further research is needed to identify what other factors would engage white collar workers to consider changing their views and drinking behaviours. All but one of the focus groups comprised male and female participants so what emerged were norms negotiated across genders – people tended not to seem as if they were excluding anyone present from the in-group. Nonetheless, gender specific focus groups with this population might be another avenue for future research, particularly given recent research which has observed gender-related socioeconomic patterning in alcohol consumption [23].

## Conclusions

The results of this study provide insight into white collar workers’ views of alcohol use. For our focus groups, the problem drinker was constructed around visibility and an inability to function, making excessive drinking the province of the ‘other’, something which was far removed from their own behaviour or that of their peers. These findings suggest that current public health interventions have not been effective in engaging this group who are likely to drink at unhealthy levels but be highly resistant to reducing their alcohol consumption - especially as they do not consider their use to be problematic unless it impairs their capacity to fulfil responsibilities or function at work. Future public health messages around alcohol should be less focussed upon the crime and personal safety implications of irresponsible drinking and be more sensitive to the lifestyles and long-term health of the populations they target.

## Competing interests

The authors declare that they have no competing interests.

## Authors’ contributions

JL conceived the study and participated in its design and coordination and drafted the manuscript. KS & GBW carried out data collection and drafted the manuscript. AC, EK & LBJ helped to draft the manuscript. CH conceived the study and participated in its design and coordination. All authors read and approved the final manuscript.

## Pre-publication history

The pre-publication history for this paper can be accessed here:

http://www.biomedcentral.com/1471-2458/12/892/prepub
